# Investigating sex differences in developmental origins of adolescent depression

**DOI:** 10.3389/frcha.2025.1602523

**Published:** 2025-08-18

**Authors:** Elizabeth C. Braithwaite, Esther Hargreaves, Jonathan Hill, Andrew Pickles, Helen Sharp, Nicky Wright

**Affiliations:** ^1^ School of Psychology, Faculty of Health and Education, Manchester Metropolitan University, Manchester, United Kingdom; ^2^ School of Psychology and Clinical Language Sciences, University of Reading, Reading, United Kingdom; ^3^ Institute of Psychiatry, Psychology and Neuroscience, Kings College London, London, United Kingdom; ^4^ Department of Primary Care and Mental Health, University of Liverpool, Liverpool, United Kingdom

**Keywords:** mis-match, maternal anxiety, adolescent depression, maternal stroking, evolutionary mechanisms

## Abstract

**Introduction:**

Evolutionary hypotheses propose that fetuses show “predictive adaptive” responses to the prenatal environment based on likely continuity with the postnatal environment, and males and females have different adaptive priorities. Female adaptations appear to implicate hypothalamic-pituitary-adrenal (HPA) axis mechanisms moderated by early tactile stimulation. Based on these hypotheses we predict that lack of prenatal-postnatal environmental continuity (mismatch), will be associated with poorer outcomes in females, an effect that will be ameliorated by tactile stimulation. We previously reported that this prediction was supported by evidence from the Wirral Child Health and Development Study (WCHADS) of a three-way interaction between maternal prenatal anxiety, postnatal anxiety, and infant stroking in the prediction of irritability at age 7 years, seen only in girls. Here, we ask whether this effect persists over another 6 years into early adolescence.

**Methods:**

Mothers in a general population cohort (WCHADS) provided self-reported anxiety scores at 20 weeks of pregnancy, and at 9 weeks, 14 months and 3.5 years postpartum, and frequency of infant stroking at 9 weeks. Their children self-reported symptoms of depression in early adolescence at age 13 years. Structural equation modelling (SEM) with maximum-likelihood estimation was conducted using data from *N* = 695 mother-child dyads.

**Results:**

There was a three-way interaction between prenatal and postnatal anxiety and maternal stroking in the prediction of early adolescent depression, seen only in girls, consistent with our previous reports. When examining self-reported depression at age 13 years, increased stroking was associated with decreased symptoms of depression in girls in the mis-match group characterised by low prenatal and high postal anxiety, but not the high prenatal and low postnatal mis-match group.

**Discussion:**

We provide preliminary novel evidence that mechanisms likely to have evolved well before the emergence of humans, contribute to the risk of adolescent depression in girls. These findings have implications for understanding developmental origins of sex differences in adolescent depression.

## Introduction

The rise in adolescent mental health problems has been a growing concern over the past two decades (1), especially in light of research demonstrating that the majority of long-term mental illnesses emerge during adolescence and early adulthood (2). For example, in the United Kingdom, approximately half of all mental health conditions begin before the age of fourteen, and seventy-five percent are present by age twenty-four (3). As well as being a significant time of life for mental health, adolescence presents an interesting phenomenon in terms of sex differences in psychopathology. These differences are widely documented both in childhood (4) and adulthood (5), with higher male psychopathology in childhood and higher female psychopathology in adulthood (6–8). This pattern reverses, seemingly during puberty, with boys, for example, experiencing nearly double the rate of mental health disorders (compared with girls) from the ages of 6–10 years, and girls experiencing just over double the rate of mental health disorders (compared to boys) between the ages of 17 and 23 (9). Existing research has clearly highlighted the sex differences in adolescent psychopathology (10–12) but specific investigations into the developmental mechanisms contributing to adolescent mental illness are limited (13). Adolescent-specific research into the biological causes of mental health problems is particularly critical because previous research has highlighted that juvenile (pre-16 years old) and adult depression, for example, may involve different pathways (14).

One area of research which may illuminate the developmental origins of adolescent mental health is the consideration of very early (*in utero*) development. The fetal programming hypothesis stipulates that there are fetal adaptations that occur *in utero* in anticipation of the postnatal environment (15). Although this hypothesis was first used to explain the relationship between low birth weight and cardiovascular disease, there is evidence that adaptations prior to birth are evident across species and exposures, reflecting a “Predictive Adaptive Response” (PAR) mechanism (16, 17). Predictive adaptive responses refer to *in utero* adaptations to best prepare the organism for the postnatal environment, and the PAR hypothesis therefore suggests that matched prenatal and postnatal environments will be associated with beneficial outcomes, whereas mis-matched pre and postnatal environments will increase risk for poor outcomes. There is also evidence that *in utero* adaptations vary by sex. The Trivers-Willard (T-W) hypothesis frames this sex-difference within an evolutionary framework, and stipulates that if maternal health during pregnancy predicts later offspring reproductive fitness, then a male predominance of births will be evident when maternal conditions are good, because healthy males compete more successfully for mates (18). On the other hand, when maternal conditions are poor, the sex ratio is reversed to avoid bearing unsuccessful males. This is consistent with the well-documented observations that male fetuses are more susceptible to preterm birth, and are also more likely to suffer the neurodevelopmental consequences of fetal insults (19). It also suggests that females have better outcomes than males following poor maternal conditions, which is consistent with evidence that the female placenta is more adaptive to adversity (19). If this effect in females originates from fetal anticipation of matched later environments (i.e., the PAR hypothesis), then a mis-match between the pre- and postnatal conditions will create vulnerability. Therefore, in combination, the PAR and T-W hypotheses lead to the prediction that prenatal risks will operate differently in males and females: in females a mis-match between the pre and postnatal environments creates vulnerability, whereas in males, poor outcomes rise incrementally from the degree of prenatal risk.

The animal research suggests that the biological mechanism that underpins this pathway in females is via hypothalamic-pituitary-adrenal (HPA) axis dysregulation. The HPA axis is the biological system which results in the release of cortisol, the main stress hormone. Dysregulation of the HPA axis is the result of reduced expression of the glucocorticoid receptor (GR), mediated by increased methylation at the promoter region of the GR gene (20). However, in rodents this mechanism can be reversed by the degree of maternal postnatal care. High levels of maternal care (indexed by licking and grooming behaviour) increased GR expression via demethylation of the promoter region of the gene, which improved HPA regulation and reduced anxiety behaviours in the offspring (21). Thus, both pre and postnatal environmental factors act on the HPA axis to influence later affective symptoms. In animals, the postnatal effects are caused by tactile stimulation, which leads to the hypothesis that similar effects might be evident in humans from maternal stroking of infants. In the Wirral Child Health and Development Study (WCHADS) cohort, mothers self-reported frequency of infant stroking at 5 and 9 weeks after birth. We showed that, in line with the animal findings, maternal stroking was associated with demethylation of the promotor region of the GR receptor gene, specifically in infants exposed to the mismatched combination of low prenatal—high postnatal depression (22). Several papers have reported on behavioural outcomes from stroking, finding that the frequency of maternal stroking modified the relationship between prenatal anxiety and infant temperament and vagal reactivity at 29 weeks of age (23), child internalizing symptoms at 2.5 years (24), and internalizing and externalizing symptoms at 3.5 years (25). The direction of effect was consistent on each occasion: the effect of prenatal risk on child outcomes was markedly reduced for mothers who reported high levels of stroking.

We further extended these findings to the prediction of child irritability at age 7 years. Irritability is a distinct phenotype of oppositional defiant disorder (ODD) and is an antecedent to adolescent depression and anxiety (26). We ensured specificity to irritability by controlling for general psychopathology “p” (27, 28). We reported a three-way interaction between maternal prenatal anxiety, postnatal anxiety and maternal stroking in the prediction of maternally reported child irritability at age 7 years. Increased maternal stroking was associated with decreased child irritability only in the two mis-matched groups (high prenatal—low postnatal anxiety, and low prenatal—high postnatal anxiety), such that the highest irritability symptoms were evident among children of mothers with mis-matched pre and postnatal anxiety who stroked their children least frequently (27, 28). This effect was seen for irritability and not headstrong symptoms, after accounting for confounders, and was not explained by more global effects on general psychopathology. Additionally, the effect was evident in girls, but not boys, as hypothesised (and pre-registered) based on the combination of the PAR and T-W theories.

In the current study we extend these findings to predictions of adolescent self-reported depression symptoms at age 13. Our specific hypotheses were as follows:

1. Self-reported symptoms of depression at age 13 will be predicted by a three-way interaction between prenatal maternal anxiety, postnatal anxiety and maternal stroking. Elevated depression symptoms will arise from a mis-match between maternal prenatal and postnatal anxiety (i.e., low-high and high-low), in combination with low maternal stroking.
2. These associations will be evident in girls but not boys.

## Methods

### Study design and sample

The participants were members of the Wirral Child Health and Development Study (WCHADS), a prospective epidemiological longitudinal study starting in pregnancy with follow-up over several assessment points during infancy, childhood and adolescence. This cohort uses a two-stage stratified design in which a consecutive general population sample (the “extensive” sample, *N* = 1,233 recruited at 20 weeks gestation) is used to generate a smaller “intensive” sample (*n* = 316) stratified by psychosocial risk, and both are followed in tandem. This enables intensive measurement to be employed efficiently with the stratified subsample, while weighting back to the extensive sample enables general population estimates to be derived. The analyses presented here were all conducted on the larger extensive sample which was identified from consecutive first-time mothers who booked for antenatal care at 12 weeks gestation between 12/02/2007 and 29/10/2008. The booking clinic was administered by the Wirral University Teaching Hospital which was the sole provider of universal prenatal care on the Wirral Peninsula, a geographical area bounded on three sides by water. Socioeconomic conditions on the Wirral range between the deprived inner city and affluent suburbs, but with very low numbers from ethnic minorities. The study was introduced to the women at 12 weeks of pregnancy by clinic midwives who asked for their agreement to be approached by study research midwives when they attended for ultrasound scanning at 20 weeks gestation. For eligibility participants were required to be aged 18 or over, English speaking and have a live singleton birth with no gross congenital abnormality.

Ethical approval for the study was granted by the Cheshire North and West Research Ethics Committee on three occasions for longitudinal data collection, on the 27th June 2006, reference number 05/Q1506/107, 7th June 2010, reference number, 10/H1010/4, and on 22nd December 2014, reference number, 14/NW/1484 and has therefore been performed in accordance with the ethical standards laid down in the 1964 Declaration of Helsinki and its later amendments. After obtaining written informed consent, the study midwives administered questionnaires, and informed consent was obtained again at later phases of the study. Of those approached by study midwives, 68.4% gave consent and completed the measures, yielding an extensive sample of 1,233 mothers with surviving singleton babies. Data were available on all 1,233 from birth records and for anxiety measures at 20 weeks of pregnancy (“20 weeks gestation”) and on 865 mothers for infant stroking at 9.3 (*SD* = 3.6) weeks (“9 weeks”). Postnatal maternal anxiety was reported by 859 mothers at 9 weeks, by 708 mothers at 14.3 (*SD* = .9) months (“14 months”), and by 710 mothers at 41.8 (*SD* = 2.3) months (“3.5 years”). Self-reported symptoms of adolescent depression were available for 695 adolescents at 13 years of age. Parents provided written consent and adolescents provided written assent for this data collection. The changes in sample sizes at each time point are attributable to sample attrition and missing data which were accounted for in all analyses.

### Measures

#### Maternal anxiety

Maternal anxiety was assessed at 20 weeks of pregnancy using the State Anxiety Scale (29), a widely used maternal self-report measure which yields total scores ranging between 20 and 80. Postnatal maternal anxiety was assessed using the same measure at 9 weeks, 14 months and 3.5 years. Cronbach's alphas across the four measurement points were between 0.79–0.86.

#### Maternal stroking

Maternal stroking was assessed by self-report using the Parent–Infant Caregiving Touch Scale (PICTS) at 9 weeks. The PICTS has 12 items covering a range of maternal touching and communication behaviours, that load on three factors of stroking, holding and affective communication (30). Four stroking items assessed how often (1 = never, 2 = rarely, 3 = sometimes, 4 = often, 5 = a lot) mothers currently stroked their baby's face, back, tummy, arms and legs. The stroking measure was administered to the intensive subsample at 5 weeks (*n* = 268), and to the extensive sample at 9 weeks (*n* = 838). Scores at 5 and 9 weeks were correlated r = 0.58, and the stroking factor showed substantial stability over the same period. In the current study, in which analyses were conducted only with the larger extensive sample, we used the 9-week maternal stroking measure. Cronbach's alpha at age 9 weeks was 0.82. As reported previously we found no association between stroking at 9 weeks and observed maternal sensitivity at 29 weeks (23) suggesting that the stroking measure assesses a distinct dimension from maternal responsivity to infant cues.

#### Adolescent depression and anxiety symptoms

The adolescent report on the Short Mood and Feelings Questionnaire (SMFQ) was used to assess depressive symptoms (31). The scale includes 13 items assessing DSM-IV symptoms of depression over the prior two weeks. Items were rated on a 3-point scale from 1 = not true to 3 = true. Internal consistency in this sample was excellent (alpha = .92)

#### Confounders

Demographic and biological risks known to be associated with prenatal stressors and child and adolescent mental health disorders were included as potential confounders, and were consistent with our previous analyses (27, 28). Variables generated at 20 weeks of pregnancy included mother's age, her cohabiting/marital status, whether she had smoked during pregnancy, and whether or not she had stayed in full time education beyond age 18. Partner psychological abuse, which was the stratifier for the intensive sample, was assessed using a 20-item questionnaire covering humiliating, demeaning or threatening utterances in the partner relationship during pregnancy over the previous year (32). The variable included in the models was defined as the highest of the partner to participant and participant to partner scores at 20 weeks pregnancy. Socioeconomic status was measured using the revised English Index of Multiple Deprivation (IMD) (33); based on data collected from the UK Census in 2001.

### Statistical analysis

**Figure 1:**
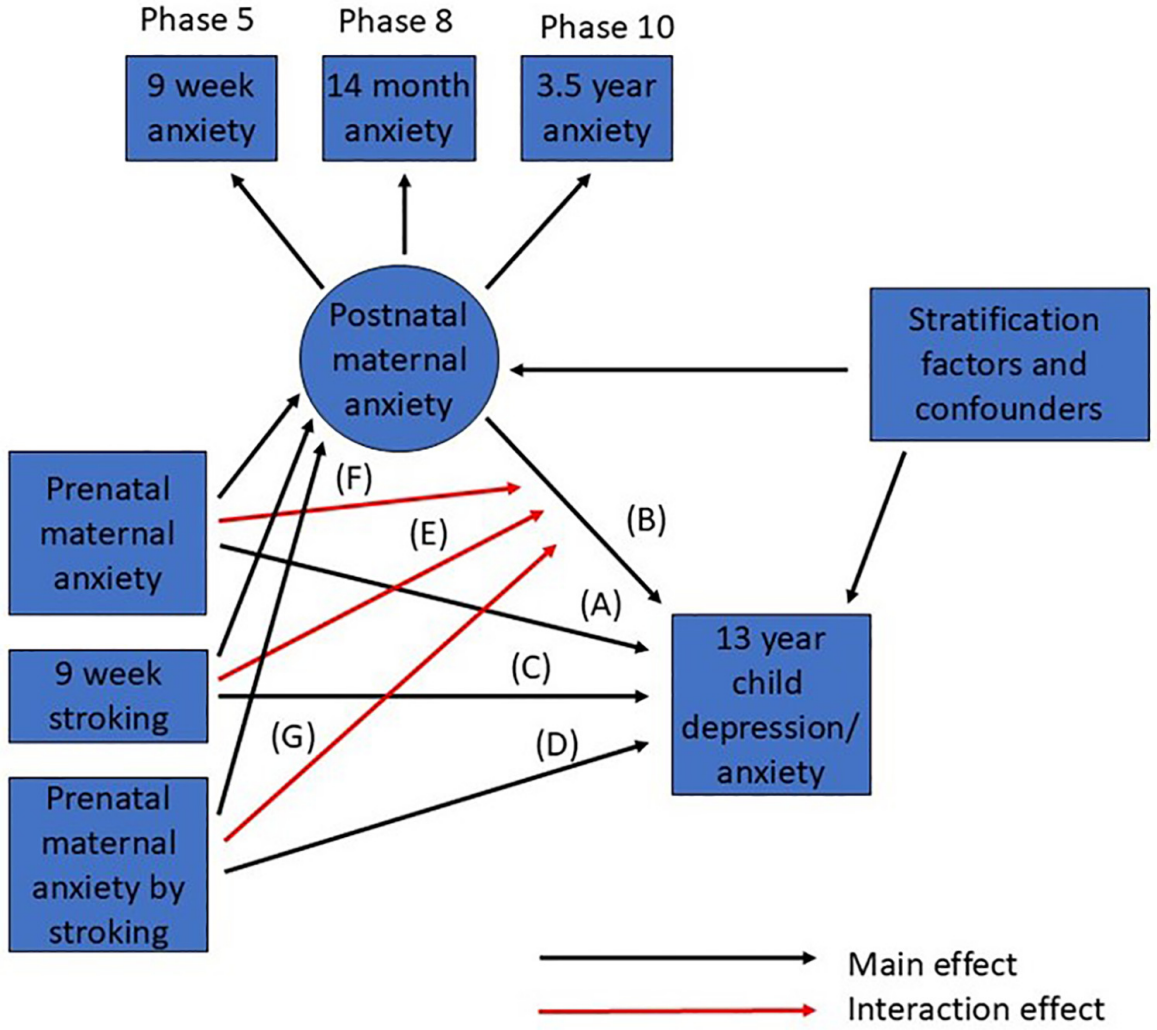
Summary of SEM for prediction of adolescent depression from prenatal and postnatal maternal anxiety and stroking. Main effects are shown in black, interactions in red. Correlations among all observed predictor (exogeneous) variables are not shown.

To best estimate long term exposure to maternal anxiety during infancy we formed a factor from the 3 postnatal measurements of maternal anxiety collected from the full cohort. Figure 1 shows the form of a series of structural equation models (SEM) that were fitted in Stata 14 to examine self-reported symptoms of adolescent depression, each square root transformed to minimise skew (correlations among all predictor variables not shown). We used the program gllamm with 15-point adaptive quadrature for model fitting (https://www.gllamm.org) (34) as this allowed us to fit all the effects shown in Figure 1 corresponding to main effects from risk factors, stratifiers and confounders (black) and interaction effects (red) that are not easily fitted in other SEM programs. This capability comes at the cost of measures of goodness-of-fit, so interpretation relies on the size and significance of estimates alone. We began by examining the estimated effects of 9-week maternal infant stroking as modifying the impact of the two-way interaction between maternal prenatal anxiety and the postnatal anxiety factor. The first model considered adolescent depression as the outcome. Estimated by using full maximum-likelihood, this model made use of data from 887 participants, who provided measures of postnatal anxiety and adolescent depression, including some with incomplete observations. The model also included the following potential confounders of the experience-behaviour relationship and predictors of participant attrition from the cohort: maternal age, smoking in pregnancy, education and marital status, and neighbourhood deprivation in addition to the stratification variables, based on partner psychological abuse at 20 weeks pregnancy. The predicted effects of matched and mismatched prenatal and postnatal anxiety were plotted using binary variables—high prenatal, low prenatal, high postnatal, low postnatal maternal anxiety—derived from median splits of the prenatal anxiety measure and the postnatal anxiety factor score. Matched groups were defined as either high prenatal-high postnatal or low prenatal-low postnatal anxiety, and mismatched groups as high prenatal-low postnatal or low prenatal-high postnatal maternal anxiety. The linear effects of maternal stroking in each of the 4 groups, and the pooled matched and mismatched groups are displayed with their corresponding regression Wald p–value for the interaction term.

## Results

Summary statistics for the study variables are shown in Table 1. Table 2 shows the estimated effects from the SEM fitted to the whole sample accounting for the stratifier and confounders, whose effects are not shown. There was a significant three-way interaction of pre- and postnatal anxiety and stroking on the prediction of adolescent depression (*p* = 0.045).

**Table 1:** Descriptive statistics for maternal and adolescent variables.

	Boys			Girls		
	N	Mean	S.D.	N	Mean	S.D.
Maternal prenatal anxiety	436	30.90	9.52	451	31.63	10.41
9 weeks maternal postnatal anxiety	424	30.40	10.12	435	29.92	9.92
14 months maternal postnatal anxiety	339	31.74	10.48	369	30.48	10.16
3.5 years maternal postnatal anxiety	345	30.40	10.80	365	30.00	10.30
9 weeks maternal stroking	436	3.88	0.70	451	3.86	0.70
Age 13 adolescent depression symptoms	319	4.03	4.48	376	7.40	6.49
Stratification stratum low	436	77%		451	75%	
Stratification stratum mid		8%			7%	
Stratification stratum high		16%			18%	
Maternal age <21 years	436	10%		451	12%	
Maternal age 21–30 years		56%			56%	
Maternal age >30 years		34%			32%	
Full time education only up to age 18	436	62%		451	67%	
Smoking—none	436	62%		451	64%	
Smoking before pregnancy		21%			19%	
Smoking during pregnancy		17%			18%	
No partner	436	17%		451	19%	
Most Deprived Quintile	436	37%		451	36%	

**Table 2:** Summary of SEM for the interaction between prenatal-postnatal maternal anxiety and maternal stroking, predicting early adolescent self-reported depressive symptoms.

	Adolescent Depression (*N* = 887 in model)	
	Coefficient (95% CI)	*p*-value
Prenatal maternal anxiety 20 weeks (A)	0.039 (0.107,0.185)	.600
Postnatal maternal anxiety factor (B)	0.456 (0.018,0.893)	.041
Maternal stroking 9 weeks postnatal (C)	−0.060 (−0.175,0.055)	0.309
Prenatal anxiety by stroking (D)	0.005 (−0.140,0.151)	.942
Postnatal anxiety by stroking (E)	−0.079 (−0.523,0.364)	.726
Prenatal anxiety by postnatal anxiety (F)	−0.166 (−0.439,0.106)	.233
Prenatal anxiety by postnatal anxiety by stroking (G)	0.266 (0.006,0.526)	.045

**Figure 2:**
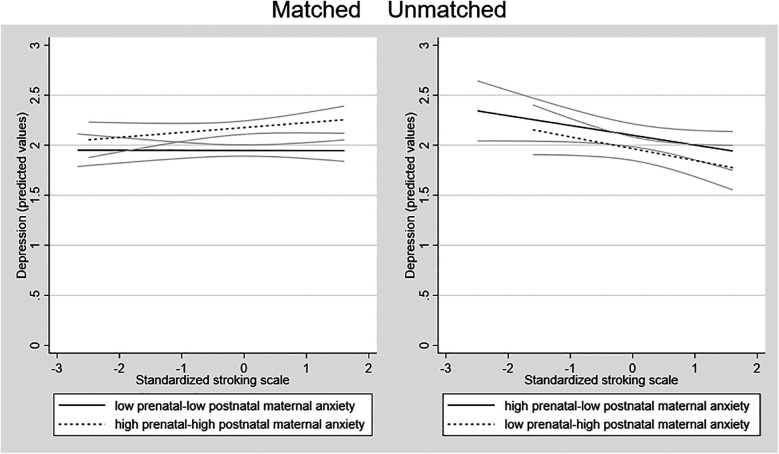
Associations between maternal stroking at 9 weeks and adolescent depression at 13 years, in matched and mismatched prenatal and postnatal maternal anxiety groups. The dotted regression lines show the association between maternal stroking age 9 weeks and depression at 13 years in each mismatched maternal anxiety group (low prenatal—high postnatal; high prenatal—low postnatal) and the solid lines in each matched group (low prenatal—low postnatal; high prenatal—high postnatal) with the 95% confidence intervals.

To examine the form of this interaction we tested the relationship of maternal stroking to adolescent depression in the four groups created by combining the binary high vs. low variables for pre- and postnatal anxiety, among which two had matching pre and postnatal conditions and two were unmatched. Consistent with the strong continuity between prenatal and postnatal anxiety, the two matched maternal perinatal anxiety groups (low–low *N* = 294, high–high *N* = 307), were larger than the mismatched maternal perinatal anxiety groups (low–high *N* = 125, high–low *N* = 161). The right-panel of Figure 2 shows that in both mismatched groups there was a negative association between maternal stroking and adolescent depression, such that the highest depression scores were predicted by a combination of maternal anxiety mismatches and low maternal stroking. In the presence of high maternal stroking, by contrast, depression scores were lower in the mismatched groups than in children exposed to persistent prenatal-postnatal maternal anxiety. There were some indications that stroking was associated with a modest increase in depression symptoms in the high-high matched group, with no effect of stroking in the low-low matched group.

**Figure 3:**
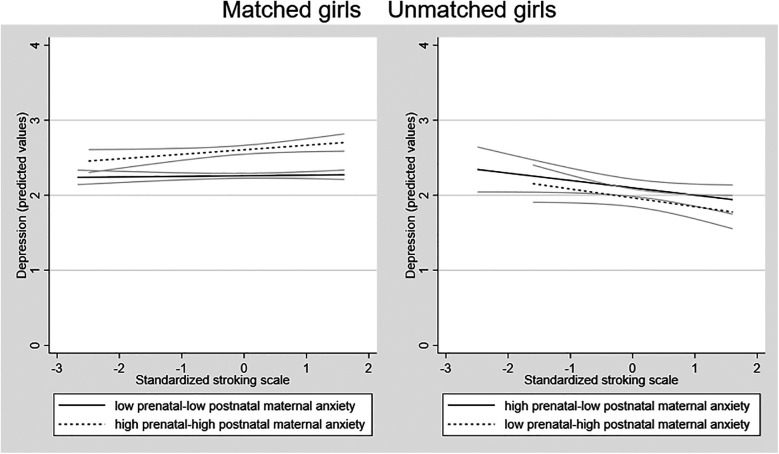
Associations between maternal stroking at 9 weeks and adolescent depression at 13 years, in matched and mismatched prenatal and postnatal maternal anxiety groups *in girls*.

**Figure 4:**
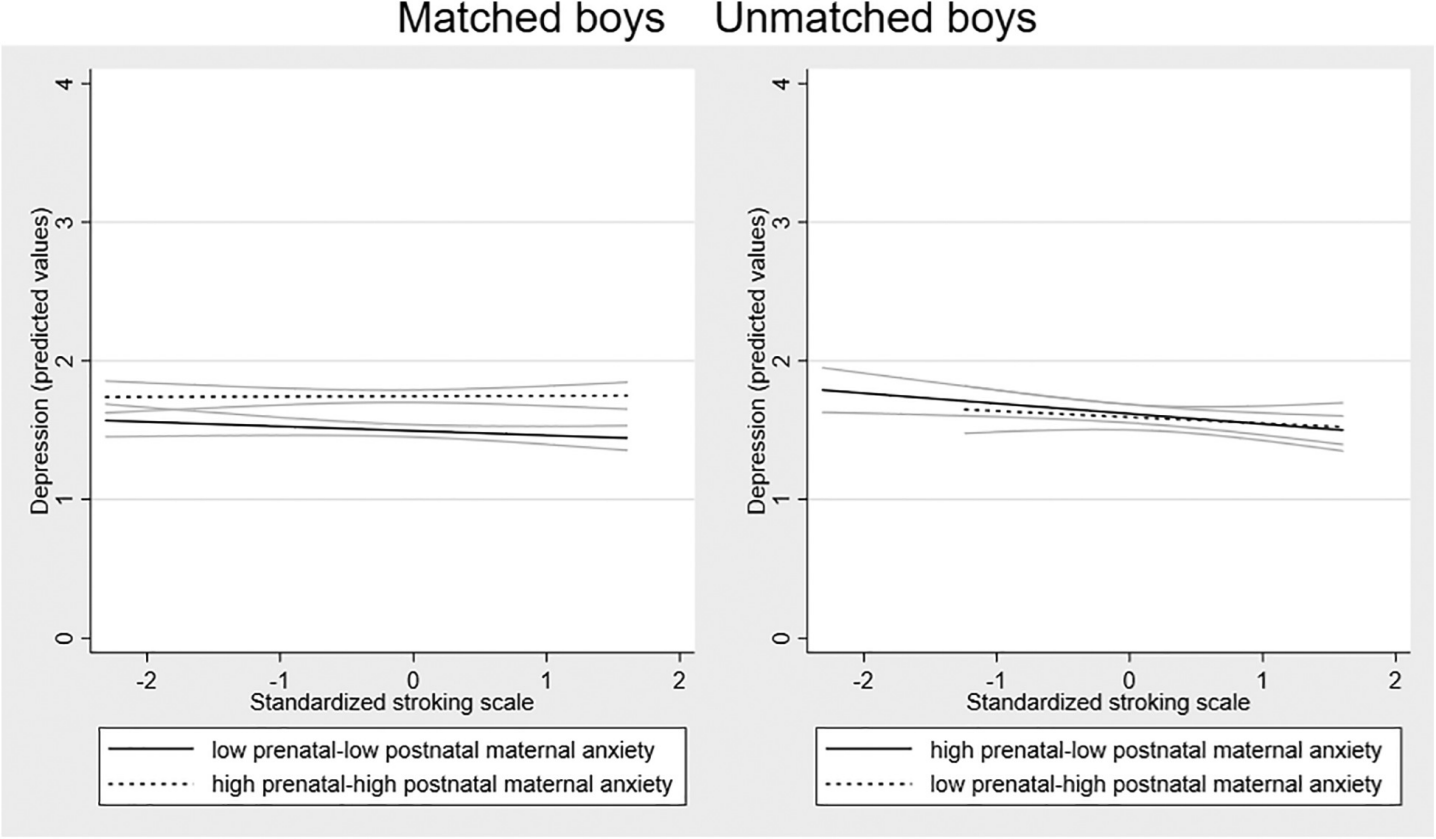
Associations between maternal stroking at 9 weeks and adolescent depression at 13 years, in matched and mismatched prenatal and postnatal maternal anxiety groups *in boys*.

Possible sex differences in the effects were examined by running the SEM models in girls and boys separately. Fitting the model to the sample of girls (*n* = 451) there was a larger 3-way interaction effect than in the whole sample [coefficient 0.356 (95% CI = 0.050 to 0.662) *p* = 0.023]. Among the boys (*n* = 436), the interaction coefficient was substantially smaller and non-significant [coefficient 0.135 (95% CI = −0.371 to 0.642), *p* = 0.601]. To examine the forms of the interactions in girls and boys, we plotted the association between stroking and adolescent depression in the four groups in girls and boys separately. Figure 3 shows that in girls there is a divergence in the two mis-match groups, with the predicted negative association between stroking and adolescent depression present in the low-high group and not the high-low group. The divergence within the matched group seen on the whole sample, with a modest increase in depression symptoms in the high-high matched group, with no effect of stroking in the low-low matched group, was also present in girls. In boys, the association between stroking and depression was similar in all the groups, see Figure 4.

We then estimated separate linear regression models to test the association between stroking and adolescent depression the matched and mis-matched groups. Given the relatively small numbers once split by sex for some of the sub-groups we focus on the variance explained for interpretation rather than *p* values. For girls, maternal stroking explained 12.8% of the variance in depression scores in the low-high mismatched prenatal-postnatal anxiety group (*p* =.052), 2% of variance in depression scores in the high-low mismatched prenatal-postnatal anxiety groups (*p* =.378) and 0% of variance in depression in the high-high (*p* = .885) and 3% low-low matched groups (*p* = .406). For boys, maternal stroking explained 5.4% of the variance in depression scores (*p* =.170) in the low-high mismatched prenatal-postnatal anxiety groups, 0% of variance in depression scores (*p* =.828) in the high-low mismatched prenatal-postnatal anxiety groups, and 1% of variance in depression in the high-high group (*p* = .697) and 2% in the low-low matched group (*p* = .697).

## Discussion

We previously developed and tested hypotheses based on two evolutionary-based theories: predictive adaptive response and the Trivers-Willard hypothesis. We demonstrated a three-way interaction between maternal prenatal anxiety, postnatal anxiety and maternal stroking in the prediction of mother reported child irritability (a precursor of depression) at age 7 years. Increased maternal stroking was associated with decreased child irritability only in the two mis-matched groups (high prenatal—low postnatal anxiety, and low prenatal—high postnatal anxiety), with the highest irritability symptoms among girls of mothers with mis-matched pre and postnatal anxiety who stroked their children least frequently (27, 28). Given that childhood irritability is an established risk factor for later symptoms of depression (35), in the current study we aimed to extend these findings to the prediction of self-reported symptoms of depression in early adolescence. Whilst replicating our previously demonstrated three-way interaction between prenatal anxiety, postnatal anxiety and maternal stroking to self-reported depression assessed in early adolescence, and in girls, this was characterised by a divergence in the mis-matched groups not previously observed. When examining self-reported depression at age 13 years, increased stroking was associated with decreased symptoms of depression in girls in the mis-match group characterised by low prenatal and high postal anxiety, but not the high prenatal and low postnatal mis-match group. Nonetheless, the core hypotheses that there is an interplay between prenatal and postnatal anxiety in line with evolutionary hypotheses which implicate HPA mechanisms, and which can be moderated by tactile stimulation, also known to act via HPA mechanisms, and that there is a sex difference, are supported over a 13-year period.

These findings advance understanding of the well-established, but poorly understood, sex-differences in child, adolescent and adult mental health disorders (4–8). It seems that in addition to the many complex social and cultural contributors to early adolescent depression, evolutionarily conserved biological mechanisms, which are observable across many species, may also contribute to the emergence of early adolescent depression in girls. However, conditions which increase risk for girls seem to run counter to straightforward concepts of low and high-risk environments. Rather, risk for girls seems to be generated from mis-matched prenatal and postnatal maternal anxiety, in combination with low levels of maternal stroking, in line with our previous findings (27, 28). In the current study, this seems to be particularly evident among the low-high prenatal-postnatal anxiety mis-matched group, but not the high-low mismatched group. It therefore seems that for the prediction of adolescent depression, it is the lack of prediction of the negative postnatal environment from the fetal environment that generates risk (in line with the predictive adaptive response theory; that organisms adapt *in utero* to the predicted postnatal environment) in combination with low tactile stimulation, rather than lack of prediction to a benign environment from a negative fetal environment. By contrast in the matched groups for maternal perinatal anxiety (both low-low and high-high) maternal stroking explained a very small amount of variation in adolescent depression symptoms.

Limitations of the study include that prenatal and postnatal anxiety, and stroking were based on maternal report and therefore may be subject to social desirability bias and maternal reports of stroking may be impacted by maternal mood. That said, there is no evidence that stroking behaviours are related to maternal mood in the WCHADS data (23). A further limitation is that the main analyses showed interactions between maternal anxiety and stroking scores without providing evidence as to whether the interactive effects occurred across the distributions. The figures were based on widely used, but arbitrary, median threshold, and so do not provide information on effects in relation to other thresholds. In particular, it cannot be inferred that the same mismatch effects would be observed in relation to higher thresholds or diagnosable anxiety disorders. A state anxiety measure was used at 20 weeks gestation to assess timing effects of state anxiety, however we did not include a measure of trait anxiety which would have enabled us to examine the role of state anxiety more precisely. Finally, our sample was not sufficiently powered to test for a four-way interaction between maternal prenatal anxiety, postnatal anxiety, stroking and sex. Instead, we utilised three-way interactions and separate models for male and females, and therefore cannot formally interpret the findings as showing a sex difference. Replication in larger and more diverse samples is therefore needed. When exploring the effects of stroking in the four matched and mis-matched groups in males and females separately the numbers were rather small which limited the power to find significant associations, although we focus on interpreting the variance explained rather than statistical significance. Strengths of the study include that the analyses were conducted on a large, general population, prospective sample over a 14-year period, that they were based on repeated measurement of postnatal exposure to maternal anxiety, and that the outcome was based on adolescent self-report which minimises shared method variance.

These findings add to the emerging and now extensive evidence from animal and human research of sex differences in associations between prenatal maternal affective symptoms and depression in offspring (36–39). More broadly, sex differences in brain circuitry likely to be implicated in depression, have been widely reported in animal studies. These include sex differences in neural responses implicated in learned helplessness and anhedonia (40). As we noted earlier many of the sex differences may be accounted for by differences in the role of the HPA axis in depression. The association between stress and HPA axis activation is mediated in the hypothalamus by corticotrophin releasing factor (CRF) which acts at the CRF receptor (CRFr). Two sex differences at the CRFr have been identified that may contribute to differences in stress reactivity (41). First, CRH causes enhanced cellular signalling in females compared to males giving rise to HPA activation at lower levels of stress. Second, in males, CRF exposure causes receptor “internalization” into the neuron thus reducing the number of receptors on the surface and hence reducing the HPA axis activation by CRF. This protective effect is not seen in females. An increasing number of studies in humans have also revealed sex differences. For example, there are consistent findings of altered amygdala resting state functional connectivity (rsFC) in adolescents and adults with depression (42) but these associations may be modified by biological sex. In a study of adolescents, amygdala rsFC with regions implicated in emotional and somatosensory processing, salience detection, and action selection was associated with internalizing symptoms in females but not in males (43).

While the predictive adaptive response phenomenon can be identified across many species, evidence in humans is more limited (44) and largely confined to reports from our group (22, 27, 28, 37). Wider replication is required before strong conclusions regarding translation can be drawn, however the findings do alert us to the possibility that low levels of maternal affective symptoms during pregnancy may create vulnerability when followed by high levels postnatally, perhaps as a result of a marked change in family circumstances, particularly for girls. More broadly our findings illustrate the utility of evolutionary theory in generating testable scientific hypotheses, with implications for our understanding of developmental processes across the lifespan.

If, as these findings suggest, there are sex differences in the biological mechanisms for depression, randomised controlled trials (RCTs) should examine for moderator effects of sex, opening the possibility of sex-dependent treatments. As an example, Arns et al. (45) found that EEG alpha asymmetry predicted outcome from anti-depressant medication, but only in females. Similarly, translation of the findings presented here, together with our previous findings, would point to the need to stratify trials, by prenatal and postnatal maternal affective symptoms, and by child sex. This is most strikingly the case for the role of maternal stroking. Interventions to promote tactile stimulation of babies have been widely advocated (46) and based on our findings, may have beneficial effects that extend into adolescence. Equally these effects may be confined to girls whose mothers’ developed depression in the postnatal period. In that case, stratification would reveal those for whom maternal stroking was most beneficial, while at the same time avoiding the risk of a null finding due to lack of effect in other groups. Studies of this kind would be enriched by an examination of potential mediators such as GR receptor gene demethylation, which we previously reported, is associated with elevated stroking following the low prenatal depression—high postnatal depression mismatch (22).

In countries such as the UK with universal antenatal and postnatal care, stratified trials of this kind, addressing questions such how do the timing, frequency and duration of tactile stimulation affect outcomes, would be relatively straightforward to establish. For the same reasons, once the evidence is clear, promotion of parental tactile simulation would be well suited to implementation as a preventative public health intervention.

## Data Availability

The data analyzed in this study is subject to the following licenses/restrictions: Due to ethical constraints supporting data cannot be made openly available. Supporting data are available to bona fide researchers on approval of an application for access. Further information about the data and conditions for access are available at the University of Liverpool Research Data Catalogue: https://doi.org/10.17638/datacat.liverpool.ac.uk/564. Requests to access these datasets should be directed to https://doi.org/10.17638/datacat.liverpool.ac.uk/564.
